# A Knock-In *Npm1* Mutation in Mice Results in Myeloproliferation and Implies a Perturbation in Hematopoietic Microenvironment

**DOI:** 10.1371/journal.pone.0049769

**Published:** 2012-11-30

**Authors:** Shiu-Huey Chou, Bor-Sheng Ko, Ji-Shain Chiou, Yueh-Chwen Hsu, Mong-Hsun Tsai, Yu-Chiao Chiu, I-Shing Yu, Shu-Wha Lin, Hsin-An Hou, Yi-Yi Kuo, Hsiu-Mei Lin, Ming-Fang Wu, Wen-Chien Chou, Hwei-Fang Tien

**Affiliations:** 1 Department of Life Science, Fu-Jen University, Taipei, Taiwan; 2 Division of Hematology, Department of Internal Medicine, National Taiwan University Hospital, Taipei, Taiwan; 3 Institute of Cellular and Systemic Medicine, National Health Research Institute, MiaoLi County, Taiwan; 4 Graduate Institute of Clinical Medicine, National Taiwan University College of Medicine, Taipei, Taiwan; 5 Institute of Biotechnology, National Taiwan University, Taipei, Taiwan; 6 Graduate Institute of Biomedical Electronics and Bioinformatics, National Taiwan University, Taipei, Taiwan; 7 Transgenic Mouse Models Core, National Taiwan University College of Medicine, Taipei, Taiwan; 8 Department of Laboratory Medicine, National Taiwan University Hospital, Taipei, Taiwan; 9 Animal Medicine Center, National Taiwan University College of Medicine, Taipei, Taiwan; Emory University, United States of America

## Abstract

Somatic *Nucleophosmin (NPM1)* mutation frequently occurs in acute myeloid leukemia (AML), but its role in leukemogenesis remains unclear. This study reports the first “conventional” knock-in mouse model of *Npm1* mutation, which was achieved by inserting TCTG after nucleotide c.857 (c.854_857dupTCTG) to mimic human mutation without any “humanized” sequence. The resultant mutant peptide differed slightly different from that in humans but exhibited cytoplasmic pulling force. Homozygous (*Npm1^c+/c+^*) mice showed embryonic lethality before day E8.5, wheras heterozygous (*Npm1^wt/c+^*) mice appeared healthy at birth and were fertile. Approximately 36% of *Npm1^wt/c+^* mice developed myeloproliferative disease (MPD) with extramedullary hematopoiesis. Those *Npm1^wt/c+^* mice that did not develop MPD nevertheless gradually developed monocytosis and showed increased numbers of marrow myeloid precursors. This second group of *Npm1^wt/c+^* mice also showed compromised cobblestone area formation, suggesting pathology in the hematopoietic niche. Microarray experiments and bioinformatic analysis on mice myeloid precursor cells and 227 human samples revealed the expression of CXCR4/CXCL12-related genes was significantly suppressed in mutant cells from both mice and humans. Thus, our mouse model demonstrated that *Npm1* mutation can result in MPD, but is insufficient for leukemogenesis. Perturbation of hematopoietic niche in mutant hematopoietic stem cells (implied by underrepresentation of CXCR4/CXCL12-related genes) may be important in the pathogenesis of *NPM1* mutations.

## Introduction

*NPM1* mutation in acute myeloid leukemia (AML) was first identified by the aberrant cytoplasmic localization of NPM1 protein, which is normally located in the nucleoli of non-mitotic cells [1]. The mutation consists of a tetra-nucleotide insertion near the C terminal end of the coding sequence of *NPM1*. The most frequent form of mutation is duplication of TCTG (type A, c.860_863dupTCTG), which results in alteration of the peptide sequence from DLWQWRKSL* to DLCL AVEEVSLRK* [1],[2]. The insertion is near the stop codon of the gene, but nonetheless disrupts 2 tryptophan residues that are critical for the localization of wild-type NPM1 protein in nucleoli [3]. Moreover, the mutant peptide contains a nuclear export signal (NES), causing the mutant NPM1 protein to shift into cytoplasm [4]. Some less frequent mutant forms involve insertion of 4 nucleotides between coding sequence position 863 and 864 or further 3′ ends; invariably, these mutations disrupt at least 1 tryptophan residue [2]. *In vitro* study has shown that an increased number of disrupted tryptophan residues is associated with incremental “traction” power of mutant NPM1 protein toward the cytoplasm [5].

The NPM1 protein is ubiquitously expressed in mammalian cells. The protein shuttles between nuclei and cytoplasm, with pleiotropic functions including ribosomal biogenesis [6], [7], centrosome duplication [8], and regulation of p53 and ARF proteins [9], [10]. Constitutional knock-out of *Npm1* in mice results in embryonic lethality [11]. Heterozygous *Npm1* knock-out produced phenotypes mimicking human myelodysplastic syndrome or malignancies of myeloid or lymphoid cells [12]. These findings suggest a haploinsufficient tumor suppressor role of Npm1 *in vivo*. However, the phenotypes of *Npm1* haploinsufficiency of differ from those seen in AML patients with *NPM1* mutation. Lymphoid malignancies and cytogenetic changes are associated with the phenotypes of *Npm1* haploinsufficiency, whereas these 2 phenomena are rarely seen in human AML patients with *NPM1* mutation [12]. This finding, together with the fairly fixed pattern of mutation in the *NPM1* gene, suggests that the mutation is likely a gain-of-function one rather than simple haploinsufficiency.

Although the clinical and biological features of AML patients with *NPM1* mutations have been well characterized [1], [13]–[18], the pathophysiological roles of *NPM1* mutation remain to be defined. In mice bearing transgenic human *NPM1* mutation, a number of animals developed myeloproliferation [19], indicating a pathological role of mutant NPM1 protein in myeloid disorders. Similarly, zebrafish bearing enforced human *NPM1* mutant expression showed an expansion of hematopoietic cells [20]. Furthermore, a mouse model of knock-in of “humanized” *NPM1* mutation (which resulted in a peptide sequence identical to the human mutation) showed expansion of the myeloid pool but shrinkage of B cells. In addition, AML phenotypes were evident in a percentage of aging mice [21]. All these animal models suggest that mutant NPM1 causes hematopoietic expansion, but is insufficeint for leukemogenesis. The pathogenetic role of *NPM1* mutation in AML awaits further clarification.

This study was the firstto document a mouse *Npm1* mutant knock-in model. In a previous study using a humanized *NPM1* mutant knock-in model [21], human mutation nucleotide sequence was directly used to replace the wild-type mouse sequence. By contrast, we inserted TCTG after coding sequence nucleotide 857 (c.854_857dupTCTG) to mimic human *NPM1* mutation. The minor difference in nucleotide sequences between humans and mice meant that a disruption in the reading frame would result in discrepancy of peptide sequences. The mutation of our model caused a shift of wild-type C terminal peptide sequence WQWRKSL* (amino acid 286–292) to LCLAVEEISLRKGFKQFEIFCLHFCNS* (amino acid 285 to 311), a pattern that differed slightly from the change observed in human *NPM1* mutation, LCLAVEEVSLRK*. Similar to the humanized *NPM1* mutant knock-in model, our homozygous *Npm1* mutant knock-in mice showed embryonic lethality. About a third of the heterozygous mutant mice (*Npm1^wt/c+^*) in our study developed fatal myeloproliferative disease (MPD) but not AML. The *Npm1^wt/c+^* mice without MPD nevertheless developed monocytosis in the peripheral blood, and increased numbers of myeloid precursors in the marrow, as they aged. Furthermore, hematopoietic stem-progenitor cells (HSPC) from the *Npm1^wt/c+^* mice exhibited defective formation of the cobblestone areas. Microarray experiments and gene set enrichment analysis (GSEA) conducted on the cells from the mice and 227 human samples showed that expression of CXCR4/CXCL12-related genes was suppressed in the mutant cells, compared with *NPM1* wild-type cells. This finding implied a perturbation of niche functions related to mouse and human *NPM1* mutation.

## Materials and Methods

### Generation of *Npm1*-TCTG knock-in mouse model

**Figure 1:**
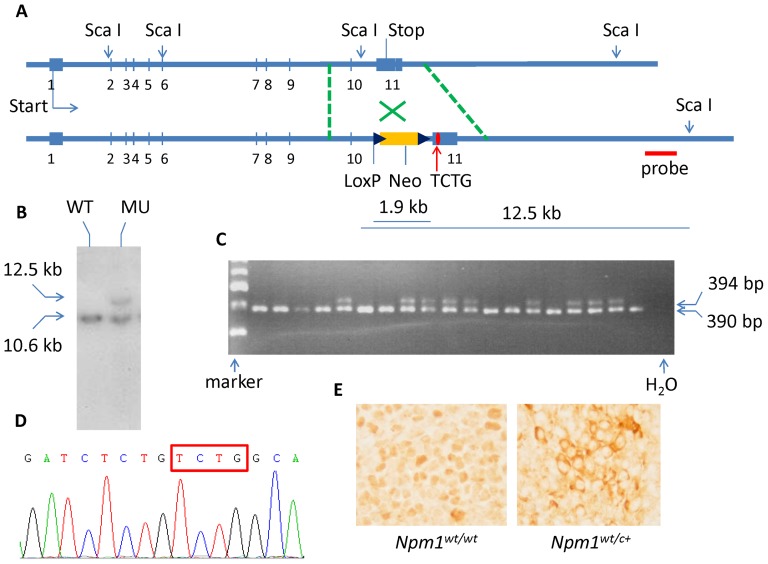
Generation of *Npm1^wt/c+^* mutation knock-in mice. (**A**) Mouse knock-in mutation construct map with the sites of ScaI restriction sites and fragment length. The loxP-neo-loxP cassette was removed before injection into foster mothers. (**B**) The knock-in was confirmed by Southern blot using a probe (as shown in panel **A**) which hybridized DNA digested with ScaI. (**C**) The mutant mice were confirmed by genotyping of the tail vein DNA. The upper band (394 bp) and lower band (390 bp) represented mutant and wild-type PCR products, respectively. Please note no homozygous mutant mice were born. (**D**) The mutant *Npm1* sequence was confirmed in DNA level by direct sequencing of the individual TA clones from PCR products of mutant mice. The box indicates the mutated nucleotides. (**E**) The immunohistochemistry of Npm1 protein in mononuclear cells obtained from mouse spleen cells. The wild-type protein is located in the nuclei, whereas the mutated Npm1 shows aberrant cytoplasmic localization.

The targeting vector was generated by retrieving whole *Npm1* gene from bMQ-282-D14 BAC clone (LifeSciences, Nottingham, UK) and subcloning into a pL253 vector; this was followed by engineering with insertion of loxP-neo-loxP cassette and replacement of the wild- type sequence with TCTG insertion after coding sequence nucleotide 857 (c.854_857dupTCTG) (Figure 1A). The targeting vectors were electroporated into cultured embryonic stem (ES) cells from 129 mouse strain. The correctly targeted ES clones were identifieded by Southern blot analysis, followed by excision of the loxP-neo-loxP cassette and implantation into foster mothers. The mice containing germ-line transmission of the TCTG-inserted *Npm1* mutant were confirmed by genotyping with PCR and sequencing of single TA clones. The PCR genotyping was performed using the following primers: forward: 5′ -GGCAACACT GGCCATAAAGT-3′; reverse: 5′ -GGTGGAGTTCCATCCTTGAA-3′. All animal protocols were approved by the Institutional Animal Care and Use Committee.

### Blood and Tissue Collection

Peripheral blood was obtained from the submandibular vein in mice at various time points up to 24 months. Blood was collected into EDTA for hematologic analysis or heparinized tubes for flow cytometry. Mouse femurs and spleens were prepared as described [22]. Briefly, sterile bone marrow (BM) was obtained by flushing the femurs and tibias with D-PBS supplemented with 2% heat-inactivated FBS using a 25-gauge needle and a 3-ml disposable syringe. Cell suspensions from the spleen tissues were made by grinding the tissues between frosted ends of microscope slides, followed by cell counting. The average number of cells in representative samples of blood, spleen, or BM under flow cytometry was used to calculate the absolute number of positive cells. All the organs and tissues were obtained postmortem under sterile conditions.

### Flow cytometery and sorting

Flow cytometric analysis of BM cells, blood, and splenocytes was performed with the following monoclonal antibodies conjugated to phycoerythrin, fluorescein, or PE-Cy5. For blood lineage analysis, we used rat anti-mouse Gr-1, F4/80, CD11b, CD3e, B220, Ter119, or NK1.1 antibodies (eBioscience, CA, USA). The hematopoietic stem-progenitor cells (HSPC), MPP (multipotent progenitor), CMP (common myeloid progenitor), GMP (granulocyte-macrophage progenitor), monocytes, and granulocytes were represented by c-kit^+^/sca-1^+^/Lin^−^, F4/80-/c-kit^+^, F4/80^+^/c-kit^+^, F4/80^+^/Gr-1^+^, F4/80^+^/Gr-1^−^, and F4/80^−^/Gr-1^+^, respectively. For marrow HSC and progenitor analyses, we used the CELLection magnetic dynabead separation kit to deplete the lineage-positive cells, and c-kit, sca-1, CD34, F4/80, or Gr-1 antibodies for staining. All data were collected and analyzed using a CyFlow instument (Partec, Germany). For collection of myeloid progenitors, a group of c-kit^+^/sca-1^−^ cells were sorted using a FACSArial sorter (BD Bioscience, CA, USA).

### Generation of *GFP-Npm1* vectors

Wild-type and mutant mouse *Npm1*- and human *NPM1*-coding sequences were fused in frame with GFP and expressed in pcDNA3.1 (Life Technologies, Grand Island, NY, USA). This was followed by forced expression in 293T-HEK cells to identify and compare the cellular localization of mutant nucleophosmin proteins from mouse and human tissues.

### Histology and immunohistochemistry

Tissue samples were fixed in PBS-buffered paraformaldehyde (4%) and dehydrated by gradual sucrose solution. Thereafter, they were embedded in Tissue-Tek O.C.T. compound medium for cryostat section. Tissues were cryosectioned at a thickness of 5 to 7 µm and used for histology or immunohistochemistry. Cryostat sections were stained with hematoxylin and eosin for histopathologic examination. For immunohistochemistry staining for Npm1 protein, we used a rabbit primary antibody (Cat. No. 3542, Cell Signaling Technology, Danvers, MA, USA).

### Hematopoietic colony-Forming Unit Assay

BM or spleen cells were plated in M3434 methylcellulose medium (Stem Cell Technologies, Canada) as described previously [23]. All cultures were repeated in triplicate, and the colonies were scored at Days 6 to 9 and Days 12 to 15 of culture. Colony types were determined by *in situ* observation using an inverted microscope.

### Cobblestone-area formation assay

To evaluate the hematopoietic differentiation and the contact behavior of hematopoietic stem and progenitor cells with marrow stromal cells, we seeded 5×10^4^ hematopoietic cells from *Npm1^wt/c+^* or wild-type mice on a confluent MS-5 BM stromal cell layer in 6-well plates. Cobblestone formation was determined after 5–6 days by counting the phase-dark cobblestone areas of at least 5 cells, using an inverted microscope. In all the experimental groups, we counted the number of cobblestone colonies as the average of 5 independent areas, each measuring 1 mm^2^ areas, using an inverted microscope.

### Microarray labeling and hybridization

Gene expression profiling was performed for both human and mouse tissues. We used Human HT-12 v4.0 BeadChip (Illumina, San Diego, CA, USA) for RNA samples obtained from mononuclear cells of BM from human samples, and we used Mouse Ref-8 BeadChip (Illumina, San Diego, CA, USA) for RNA samples from sorted mouse c-kit^+^/sca-1^−^ cells (with myeloid progenitors including MPP, CMP, and GMP). Because of limitation of the cell number, we pooled RNA from 3 mice each of either wild-type or non-MPD mutant mice for one single pair of microarray experiment. High quality RNA weighing 500 ng was converted to double-stranded cDNA, followed by an amplification step (*in vitro* transcription, Ambion, Austin, TX, USA) to generate biotin labeled cRNA (Ambion). Subsequently, 1.5 µg cRNA was hybridized to BeadChips at 58°C. After 14–20 hours hybridization, the BeadChips were stained by Cy3 and were then washed. The intensities of the bead fluorescence were detected by the Illumina BeadArray Reader, and the results were analyzed and quantile-normalized using GenomeStudio v2010.1 software. This study has been approved by the Institutional Review Board (IRB) of the National Taiwan University Hospital. Written informed consents were obtained from the human participants who provided tissue samples, in accordance with the regulations of the IRB.

### Gene set enrichment analysis

We used GSEA software v2.07 (www.broadinstitute.org/gsea/index.jsp) to study specific groups of genes. We were interested in 22 gene sets related to HSPC functions, which we selected from the database of Ingenuity Pathway Analysis (IPA, Ingenuity Systems, www.ingenuity.com) (Table S1). The method determines whether predefined sets of genes are enriched in the observed gene expression profile. Genes are ranked according to the fold-change, or significant differences in expression, observed among phenotypes in the whole-genome expression data. Given a predefined gene set, an enrichment score (ES) is calculated to measure the overrepresentation of members of that gene set appearing at the extremes (top or bottom) of the ranked gene list. The ES is then evaluated for significance using phenotype-based or gene-based permutation tests. The ES, together with the permutation *P* value, indicates the degree to which the defined gene set is enriched in the gene expression data. Parameters and settings used in the analyses are shown in Table S2. The gene sets include (i) 2 sets of validated up-stream regulators and down-stream targets of CXCR4 and CXCL12 respectively, and (ii) 20 groups of genes involved in cellular functions in HSPCs, of which (i) and (ii) were exported from the database of IPA. Table S1 shows the complete contents of these 22 gene sets.

### Statistical analysis

All data are presented as means ± S.E.M., and the differences between groups were assessed using the Student t test or one-way ANOVA with a significance level of *P*<0.05.

## Results

### In vivo expression of conventional *Npm1^wt/c+^* mutation in mice

To study the role of *Npm1* mutation in hematopoiesis and leukemogenesis *in vivo*, we inserted TCTG after nucleotide c.857 of murine *Npm1* coding sequence (c.854_857dupTCTG), because this pattern mimics human *NPM1* mutation but without any “humanized” sequence (Figure S1). This mutation causes a shift of peptide sequence from WQWRKSL* (amino acid 286–292) to LCLAVEEISLRKGFKQFEIFCLHFCNS* (amino acid 285 to 311), a pattern that differs slightly from the change in human *NPM1* mutation. Nonetheless it also harbors a canonical NES, [LIVFM]–x(2,3)–[LIVFM]–x(2,3)–[LIVFM]–x–[LIVFM], and is predicted to generate an NES according to NES predictor (NetNES) [24]. We first examined the localization of this putative mutant Npm1 protein by forced expression of the mutant protein that was fused with GFP protein in 293T-HEK cells. The localization of the mutant protein in cytoplasm, compared with the restricted localization of wild-type Npm1 protein in the nucleoli, was similar to human condition (Figure S2). The process of generating heterozygous mutant mice (*Npm1^wt/c+^*) is shown in Figure 1A. *Npm1^wt/c+^* genotype in our mice was confirmed by Southern blot and sequencing (Figure 1B–D). The subcellular distribution of Npm1 in blood cells from heterozygous mice was then confirmed by immunohistochemistry stain, which showed that the wild type protein was mainly located in the nuclei, whereas the mutated Npm1 protein had appeared in cytoplasm in spleen sections (Figure 1E). Homozygous *Npm1* (*Npm1^c+/c+^*) mutation resulted in embryonic lethality before E8.5 day, while *Npm1^wt/c+^* mice survived and were fertile.

### *Npm1^wt/c+^* mice showed myeloproliferative features

**Figure 2:**
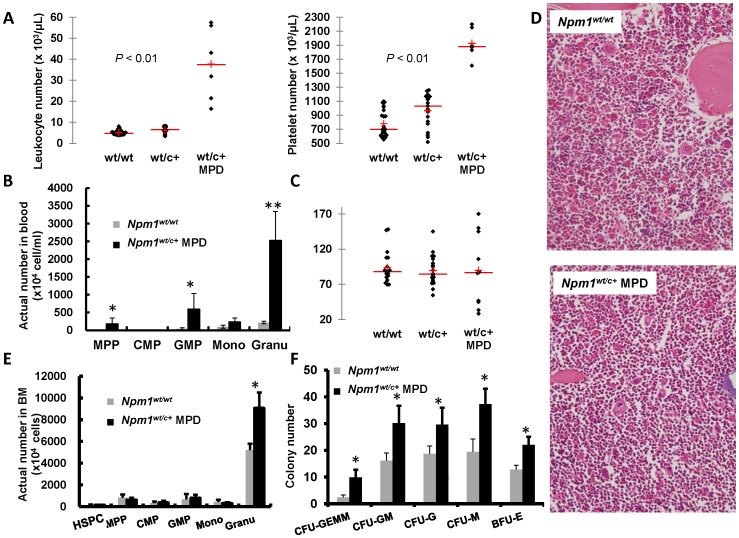
Hematopoietic changes in peripheral blood and BM in *Npm1^wt/c+^* mice with myeloproliferative features. (**A**) Monthly blood counts in peripheral blood were monitored in 24 *Npm1^wt/wt^* and 30 *Npm1^wt/c+^* mice, respectively. Five, five, and one *Npm1^wt/c+^* mice had abnormal increase of leukocytes, platelets or both in blood, respectively. (**B**) The *Npm1^wt/c+^* mice with myeloproliferative features showed higher numbers of MPP (F4/80^−^/c-kit^+^), GMP (F4/80^+^/Gr-1^+^) progenitor cells, and granulocytes, compared with wild-type mice; n = 3 in each group. (**C**) No differences of total BM cells among the mice whether they are *Npm1-*wild type or *Npm1-*mutant with myeloproliferative features or without (n = 20 for wild-type; n = 20 for *Npm1^wt/c+^* without MPD; n = 10 for *Npm1^wt/c+^* with MPD). (D) Histologic sections of BM from wild-type and MPD mutant mice did not show obvious difference in cellularity (H & E staining, 400×). However, the mature granulocyte number is significantly higher for mutant mice with myeloproliferation ; n = 3 each for wild-type and *Npm1^wt/c+^* with MPD (**E**). In addition, the colony forming ability is significantly elevated in those mutant mice with myeloproliferation; n = 4 each for wild-type and *Npm1^wt/c+^* with MPD (**F**). * means *P*<0.05; ** means *P*<0.01. MPP, multipotent progenitor; CMP, common myeloid progenitor; GMP, granulocyte-macrophage progenitor; CFU-GEMM, CFU-granulocyte, erythroid, macrophage, megakaryocyte; CFU-GM, CFU-granulocyte macrophage; CFU-G, CFU-granulocyte; CFU-M, CFU-macrophage, and BFU-E, burst-forming unit-erythroid.

Data from mice aged between 2 to 24 months were analyzed. Eleven mice among 30 (36%) *Npm1^wt/c+^* mice developed either leukocytosis (n = 5), thrombocytosis (n = 5), or both (n = 1); this pattern mimicked MPD in humans (Table 1). The 11 *Npm1^wt/c+^* MPD mice displayed significantly higher platelet counts or white blood cell counts (*P*<0.01) compared with the age-matched wild-type or *Npm1^wt/c+^* mice without MPD (Figure 2A). The red cell volumes, red blood cell count, hemoglobin, and mean platelet volume did not differ between the groups (data not shown). In *Npm1^wt/c+^* mice with MPD, leukocytosis appeared at various ages, whereas thrombocytosis occurred only late in life (Table 1). Several mice displaying myeloproliferative features were fatally affected, but others were not moribund at the time of sacrifice. Flow cytometric analysis of blood cells showed a significant increase in the number of mature granulcoytes in *Npm1^wt/c+^* mice with MPD (Figure 2B). Specifically, a group of hematopoietic progenitors including multipotent progenitors (MPP) (F4/80^−^/c-kit^+^) and granulocyte-macrophage progenitors (GMP) (F4/80^+^/Gr-1^+^), were detected in blood taken from the mice (Figure 2B). Although the total bone marrow (BM) cell count and cellularity in histopathology section did not differ significantly between the groups (Figure 2C and 2D), a significant higher count of mature granulocytes in BM was observed in mutant mice having myeloproliferative features (Figure 2E). Also, marrow cells from these mutant mice showed an increase in myeloid and erythroid colony formation (Figure 2F).

**Table 1:** The hematopoietic changes in white blood cell count, platelet count, and hemoglobulin of peripheral blood in wild type, *Npm1^wt/c+^* mice without signs of myeloproliferative disease (MPD), and *Npm1^wt/c+^* mice with sign of MPD.

	n	WBC (10^6^/mL)	PLT (10^6^/mL)	Hb (g/dL)	Age of MPD^a^
*Npm1^wt/wt^*	24^b^	5.15±0.22^d^	805.42±42.84	13.26±0.44	
*Npm1^wt/c+^*	19^b^	5.91±0.31	967.52±49.14	12.92±0.29	
*Npm1^wt/c+^* MPD	11	23.95±6.06	1518.27±153.77	11.55±0.683	
number^c^	#5	5.2^e^	1610	13.5	22 mon.
	#7	10	2153	11	22 mon.
	#9	21.45	1295	14.4	24 mon.
	#18	56	1048	12.8	22 mon.
	#17	7.5	1891	11	22 mon.
	#24	43.1	1061	9.9	6 mon.
	#36	4.3	2200	12	22 mon.
	#54	10	1831	8.5	22 mon.
	#57	16.5	1874	13.8	24 mon.
	#97	57.5	1017	8.2	9 mon.
	#66	31.9	721	12	12 mon.

a Age of MPD: the age when MPD developed in *Npm1*-mutated mice.

b The leukocytes (WBC), platelets (PLT), and hemoglobulin (Hb) count of peripheral blood from wild type and mutant mice without MPD were collected at 12 months.

c number: the code representing each *Npm1^wt/c+^* mouse with sign of MPD.

d Data is represented as mean ± SE.

e Data is represented as individual value of each *Npm1^wt/c+^* mouse with MPD.

**Figure 3:**
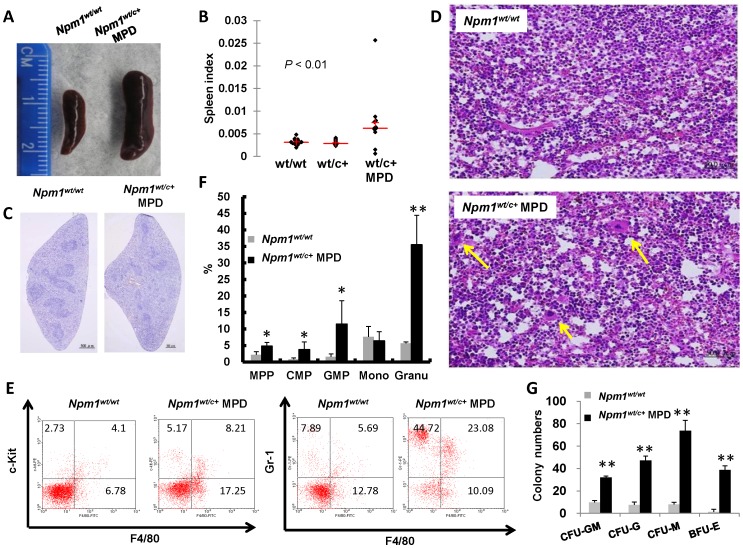
Hematopoietic changes of spleen in *Npm1^wt/c+^* mice with myeloproliferation. (**A, B**) Mutated *Npm1^wt/c+^* mice with signs of myeloproliferation had splenomegaly and higher spleen weight index (spleen weight/body weight). n = 20 for wild-type; n = 20 for *Npm1^wt/c+^* without MPD; n = 10 for *Npm1^wt/c+^* with MPD). (**C, D**) Spleen sections from wild-type and MPD mutant mice stained with hematoxylin demonstrated changes in the follicle structure (C), and infiltration with megakaryocytes (arrows), indicating extramedullary hematopoiesis (D). (**E**) *Npm1^wt/c+^* mice with signs of myeloproliferation had more CMP (c-kit^+^/F4/80^+^, right upper quadrant of the left panel), GMP (Gr-1^+^/F4/80^+^, right upper quadrant of the right panel), and granulocytes (Gr-1^+^/F4/80^−^, left upper quadrant of the right panel), as shown by flow cytometry. (F, G) *Npm1^wt/c+^* mice with signs of myeloproliferation had extramedullary hematopoiesis as shown by increased immature and mature myeloid cells (n = 3 each; * *P*≤0.05) and hematopoietic colony formation in spleen cells (n = 3).

The *Npm1^wt/c+^* mice with MPD exhibited splenomegaly and higher spleen-weight index compared with wild-type or non-MPD mutant mice (Figure 3A–B). Histomorphological examination of the *Npm1^wt/c+^* mouse spleens showed effacement of follicle structure, and extramedullary hematopoiesis with megakaryocyte infiltration (Figure 3C–D). Flow cytometric analysis and colony formation assay confirmed the existence of hematopoietic precursor cells and panmyelosis in the spleens (Figure 3E–G).

### *Npm1^wt/c+^* mice without MPD revealed myeloid expansion

**Figure 4:**
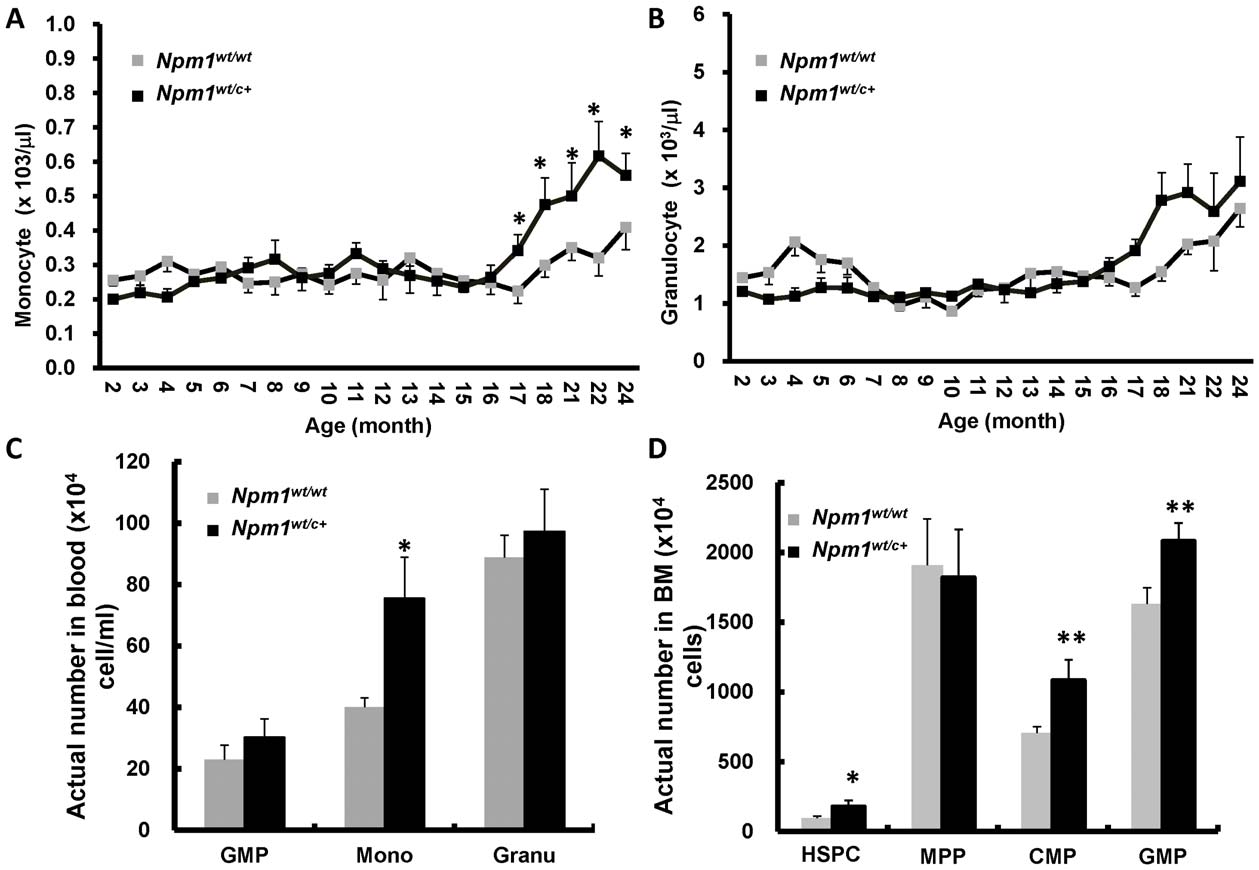
The hematologic changes of peripheral blood and BM in *Npm1^wt/c+^* mice without signs of myeloproliferation. Monthly blood collection from wild-type and *Npm1^wt/c+^* mice without signs of myeloproliferation showed increase in the compartment of monocytes (**A**) but not granulocytes (**B**), lymphocytes, or platelet (data not shown). Each data point was the average of at least 10 mice. (**C**) There were significant more monocytes (F4/80^+^/Gr-1^−^) in non-myeloproliferative *Npm1^wt/c+^* mice compared to wild-type mice at age of 18 months (n = 7 each). (**D**) At age of 12 months (n = 7 each), quantification of marrow cells of HSPC (c-kit^+^/sca-1^+^), MPP (F4/80^−^/c-kit^+^), CMP (F4/80^+^/c-kit^+^), GMP (F4/80^+^/Gr-1^+^), monocytes (F4/80^+^/Gr-1^−^), and granulocytes (F4/80^−^/Gr-1^+^) by flow cytometry revealed significant increase in marrow stem cell and progenitor cell compartments of HSPC, CMP, and GMP, but not MPP. Data are expressed as mean ± SE. * means *P*<0.05; ** *P*≤0.01.

Most *Npm1^wt/c+^* mice obtained normal hemograms and appeared clinically disease-free throughout their lives. However, these mice developed a delayed-onset increase of monocytes (but not granulocytes, lymphocytes or platelets) in their peripheral blood after the age of 17 to 18 months (Figure 4A–B, and data not shown). Flow cytometric analysis of blood cells confirmed an increased proportion of mature monocytes (F4/80^+^/Gr-1^−^) in 18-month-old *Npm1^wt/c+^* mice without MPD (Figure 4C). These BM of these mice also exhibited expansion of the myeloid compartment, with an increased number of HSPC (c-kit^+^/sca-1^+^), as well as progenitors (CMP, c-kit^+^/F4/80^+^ and GMP, F4/80^+^/Gr-1^+^) (Figure 4D).

### Compromised cobblestone area formation in BM cells of *Npm1^wt/c+^* mice

**Figure 5:**
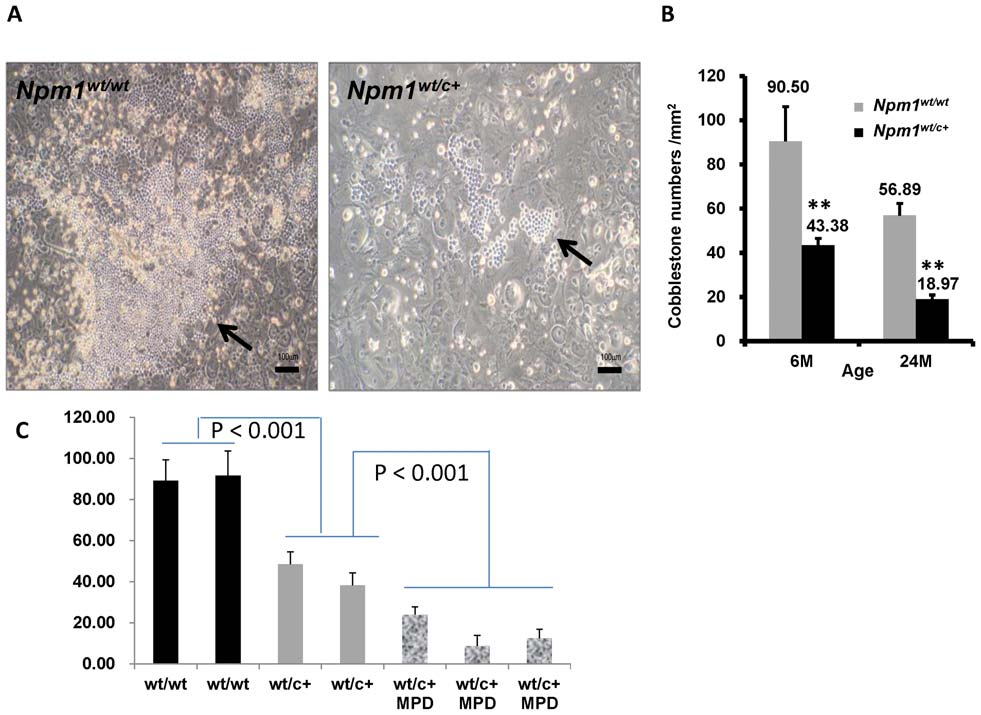
Age-dependent decline of cobblestone area formation in *Npm1^wt/c+^* mice. (**A**) The cobblestone area formation (arrows) is obviously inhibited in *Npm1^wt/c+^* HSPC. (**B**) The cobblestone area numbers are also much lower in mutant mice. The difference seems more augmented as mice became older (n = 7 each). (C) The mutant mice with MPD exhibited even more severe compromised cobblestone area formation when compared with mutant but non-MPD mice (*P*<0.001, one bar represents triplicate experiments from one mouse).

The majority of mutant mice did not develop clinically evident MPD. However, the gradual development of monocytosis and increased numbers of myeloid precursors in these mice might imply the presence of a pathological hematopoietic niche in BM. The extramedullary hematopoiesis observed in the MPD mice suggests the same possibility. We thus examined the effect of *Npm1^wt/c+^* mutant cells on the interaction of HSPCs and marrow stromal cells by *ex vivo* cobblestone area formation assay. We collected 5×10^4^ hematopoietic cells from *Npm1^wt/c+^* mice without MPD, and from wild-type mice of various ages; the collected cells were placed on the MS5 stromal cell layers for cobblestone-like colony formation. The mutant cells exhibited severely compromised cobblestone area formation compared with wild-type cells (Figure 5A). In 6-month-old *Npm1^wt/c+^* mice without MPD, a 2-fold decrease in cobblestone area numbers was noted, and by 24 months the decrease was 3-fold compared with wild-type mice (Figure 5B). These findings imply abnormalities in the contact signal pathways of mutant HSPC in the context of the microenvironment. The same defect was seen and even more evident in mutant mice with MPD (Fig. 5C).

### Correlation between *Npm1* mutation and expression of CXCL12/CXCR4 pathway genes

**Figure 6:**
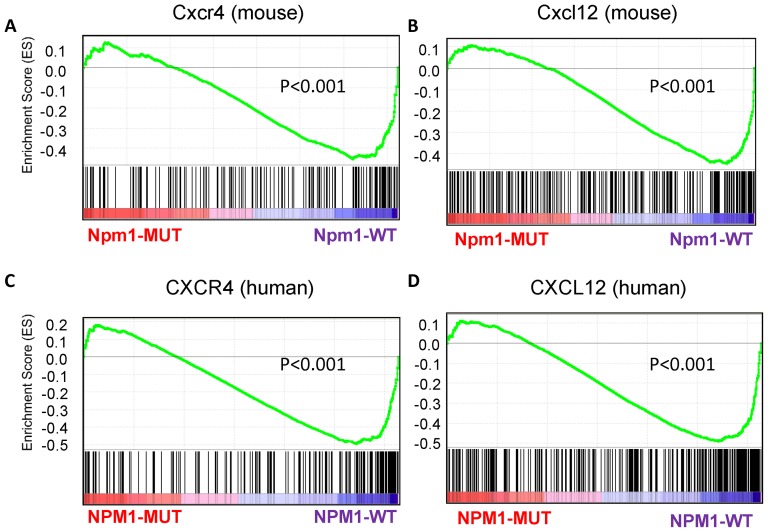
Enrichment plots from gene set enrichment analysis (GSEA). The enrichment plots contain profiles of the running enrichment scores (ES) and positions of gene set members on the rank ordered list in GSEA. (**A**) Cxcr4 and (**B**) Cxcl12 interaction signatures on the mouse data. (**C**) CXCR4 and (**D**) CXCL12 interaction signatures on the human data.

To explore the mechanisms underlying the defective cobble stone area formation, we compared the gene expression profiles of sorted BM myeloid progenitors (c-kit^+^/sca-1^−^/F4/80^−^) between wild-type and *Npm1^wt/c+^* mice without MPD. We conducted GSEA to explore pathways that might be responsible for defective cobblestone area formation in *Npm1^wt/c+^* HSPC. We compared the degree of enrichment across 22 gene sets between *Npm1^wt/c+^* and wild-type expression profiles; these gene sets were related to HSPC functions, and were selected from the IPA (Table S1). Among the gene sets, only pathways related to Cxcl12 and Cxcr4 were significantly downregulated in *Npm1^wt/c+^* mouse (both *P*-value<0.001, normalized) (Table S3 and Figure 6A–B for enrichment plots). These pathways both imply interaction between HSPCs and the marrow niche.

### Concordant suppresion of CXCL12 and CXCR4 pathway signatures in *NPM1*-mutated AML patients

To investigate whether differential enrichment of CXCL12/CXCR4 pathway-related genes occurs in human AML, we performed genome-wide expression microarray analysis on BM mononuclear cells. The sample cells were collected from a cohort of 197 AML patients who exhibited either 147 *NPM1*-wild-type or 50 *NPM1*-mutated, and 30 normal BM transplantation donors. The GSEA showed obvious suppression of CXCL12 and CXCR4 pathway genes in *NPM1*-mutated patients compared to *NPM1*-wild AML and normal controls (normalized *P*-values of both <0.001, Table S3; enrichment plots, Figure 6C–D). The results of the GSEA are shown in Table S4. In addition to measuring the degree of enrichment, GSEA identified a leading edge subset in each gene set, comprising core gene members that contributed the most to the enrichment scores. The leading-edge members represent genes that might participate in biological functions governed by that specific gene set in the observed data (Table S5).

## Discussion

The earliest report of *NPM1* mutation in AML was published 2005 [1], but to date the molecular mechanisms underlying the pathogenesis are not yet well understood. Apart from this study, 3 other animal models (including transgenic mouse and zebrafish models [19], [20] and a “humanized” knock-in mouse model [21]) have been generated and analyzed. All of these studies have used human mutation sequences, but ours was the first to use an “endogenous” mouse sequence, which we named “conventional” mouse model. The mutated peptide sequence in our mutant mice differed from that in humans because of a discrepancy in the nucleotide sequence of the gene in mice versus humans. Nonetheless, we showed that cellular localizations of mutated and wild-type Npm1 proteins were different. However, the phenotypes of our mouse model were not identical to those present in the previous 2 mouse studies. In the transgenic mouse model, mice developed non-fatal myeloproliferation in the BM and spleen, but did not show leukocytosis in peripheral blood [19]. In addition, 30% of the “humanized” knock-in mice showed expansion of mature myeloid cells and late-onset AML [21]. By contrast, in our “conventional” mouse model, approximately one third of the mutant mice showed myeloproliferation at various ages, but no AML was seen throughout their lifetimes. The phenotypic discrepancy between the mice in our study and those in the transgenic model can be explained by their use of exogenous promoter to drive human *NPM1* mutant gene in the presence of full-dose wild-type mouse Npm1 protein. The differences of the phenotypes between our mice and the “humanized” knock-in model are likely related to different mutation sequences. Although both mutant proteins can be localized in cytoplasm, the strength of cytoplasmic retention strength may differ in the 2 proteins, which would result in different nucleus-to-cytoplasm ratios for Npm1 protein. Alternatively, the extra amino acids observed after the NES in our study might have led to unexpected consequences in cells. Nevertheless, all the models suggest a role of mutant nucleophosmin protein in aberrant myelopoiesis. Overall, all the models suggest that *NPM1* mutation alone does not provide sufficient conditions for AML formation. Other pathological abnormalities are necessary to induce full-blown leukemia.

Although in human, MPD is usually associated with increased marrow cellularity, splenomegaly, and peripheral blood cytosis, there is occasional inconsistency among these organs/tissues in mice. For example, in a report, the MPD was shown in BM and spleen but not PB [19]. In another study where a myeloproliferative syndrome was induced in mice, elevated myeloid cells were seen in spleen and blood, but BM showed paradoxically reduced cellularity [25]. We did not see difference in the BM cellularity or precursor cell number among the wild type, non-MPD mutant, and MPD mutant mice. It is likely that the origin of MPD may arise from extramedullary organs, such as spleen, related to extramedullary hematopoiesis.

Is the defective cobblestone area formation, which reflects the repopulating ability of HSPC, in *Npm1*-mutated mice truly microenvironment-dependent or cell-intrinsic? To address this question, we have to clarify two points: first, did the cells used in the cobblestone assays contain the same number of progenitor cells (which are supposed to be the source of cobblestone colonies) between wild-type and mutant mice? To answer this point, we seeded equal number of Lin- progenitor cells on murine stromal cells and found that the colony numbers in wild-type cells were still significantly greater than mutant cells (data not shown). Secondly, do the progenitor cells from wild-type or mutant mice grow at the same speed without stromal cells? We noted that the CFU-GEMM and CFU-GM numbers were not different between wild-type mice and non-MPD mutant mice at 12 months of age (data not shown). Therefore, it seems that the proliferation of the progenitor cells from these 2 groups of mice differ only when stromal cells are added for co-culture. Therefore, these results suggest that the decreased cobblestone colony number in mutant cells is likely related to microenvironment rather than intrinsic cell factors. However, further investigations are warranted to get more definitive answers.

To investigate the possible mechanisms underlying this phenomenon, we employed GSEA tool, which is a powerful computational tool for interpreting whole-genome gene expression data at the level of gene sets [26], [27]. The main advantage of GSEA over the traditional strategies of examining differentially expressed genes is the capacity to detect overall trends and differences throughout the entire network. In addition, threshold-free analysis enables the identification of gene-set level enrichment when the underlying changes in individual genes are modest and subtle. Studied gene sets can include built-in gene sets of the Molecular Signature Database [27], including functionally defined groups (e.g., Gene Ontology terms), canonical pathways (e.g., BioCarta and KEGG), and signatures of chemical perturbations. Alternatively, user-defined sets of genes that share common features of interest may be studied. The prowess and flexibility of GSEA have resulted in this software being widely used in genomic data analyses [28]–[32].

The results of our study showed that expression of CXCR4/CXCL12-related genes was significantly downregulated in *Npm1^wt/c+^* mouse BM precursor cells and in human AML samples displaying the *NPM1* mutation. These genes are critical in maintaining the, HSPC pool in the BM niche [33]. Downregulated expression thus suggests an aberration in the hematopoietic niche, which might be related to abnormal interaction between these cells and stromal cells. This type of microenvironment perturbation has been implicated in human diseases such as primary myelofibrosis [34], [35]. The extramedullary hematopoiesis and myeloid expansion observed in our mutant mice mimicked the clinical features of idiopathic myelofibrosis and other myeloproliferative neoplasms. These diseases might be related to a pathological hematopoietic niche and enhanced mobilization of the HSPC to alternative niches, such as liver and spleen [34], [35]. This niche pathology worsened as our mutant mice aged. In *Npm1^wt/c+^* mice without MPD, the gradual development of monocytosis and increased numbers of myeloid precursors with aging, combined with defective cobblestone area formation, may be early signs of MPD. As-yet-unidentified genetic hits might be required to accelerate the development of clinically evident MPD for these mice.

The aging-related decline of cobblestone area formation coincides with the fact that *NPM1*-mutation AML is more prevalent among older than younger patients [1], [18]. We surmise that additional hits of genetic abnormalities (which are more likely to occur in older people) interact with the age-dependent perturbation of the hematopoietic niche to result in clinical *NPM1*-mutated AML among older patients. High CXCR4 expression in AML cells is an independent unfavorable prognostic factor [36]. A recent study showed that a CXCR4 antagonist, plerixafor, increased the sensitivity of leukemia cells to chemotherapy [37]. Previous reports have documented a similar increase in certain patients' responsiveness to chemotherapy [1], [13]. The possibility that defective CXCR4/CXCL12 interaction in AML patients bearing an *NPM1* mutation might contribute to this increased responsiveness deserves further investigation.

In conclusion, this study presented a “conventional” knock-in model of *Npm1* mutation. The main phenotypes were myeloproliferative neoplasms with extramedullary hematopoiesis, which occurred in approximately 30% of the mutant mice. Other non-myeloproliferative *Npm1^wt/c+^* mice showed delayed onset of monocytosis in the peripheral blood and increased numbers of BM myeloid precursors as the animal aged. The results of *ex vivo* cobblestone assay implied a perturbation of the hematopoietic niche in mutant mice from as early as 6 months of age. This finding might be related to defective CXCR4/CXCL12 pathways. This hypothesis was supported by genome-expression data from a large cohort of human samples. The findings of our study provide further evidence that *NPM1* mutation alone is insufficient to cause AML formation. The relatively suppressed expression of CXCR4/CXCL12 pathway genes, observed in our mutant mice and *NPM1*-mutated AML patients, might be critical in the pathogenesis of *NPM1* mutation.

## Supporting Information

Figure S1 **The alignment of nucleotide and protein sequences of wild-type and mutant NPM1 of human and mice.** CDS, coding sequence.
(TIF)

Figure S2 **The cytoplasmic localization of mutated nucleophosmin protein from human and mice.** Upper panel: GFP fused at the N terminal end of human wild-type NPM1 (left) localizes mainly in the nucleoli as punctate pattern. In contrast, GFP fused with human mutant NPM1 protein mainly resides in cytoplasm, although some appears in the nucleoli. Lower panel: GFP fused at the N terminal end of wild-type mouse Npm1 protein (left) and expected mutant Npm1 protein (right) exhibit similar patterns as the human condition.
(TIF)

Table S1 **Updated defined gene sets for GSEA.**
(XLS)

Table S2 **Parameters and settings of GSEA in this Study.**
(DOCX)

Table S3 **Summary of mouse and human gene expression data in gene set enrichment analysis.**
(DOCX)

Table S4 **Complete summary of GSEA.**
(XLS)

Table S5 **Leading-edge genes from GSEA.**
(XLSX)
