# Supplementary figures and images for: A Knock-In Npm1 Mutation in Mice Results in Myeloproliferation and Implies a Perturbation in Hematopoietic Microenvironment
Source: PLoS One. 2012 Nov 30;7(11):e49769. doi: 10.1371/journal.pone.0049769 (PMC3511491; doi:10.1371/journal.pone.0049769)

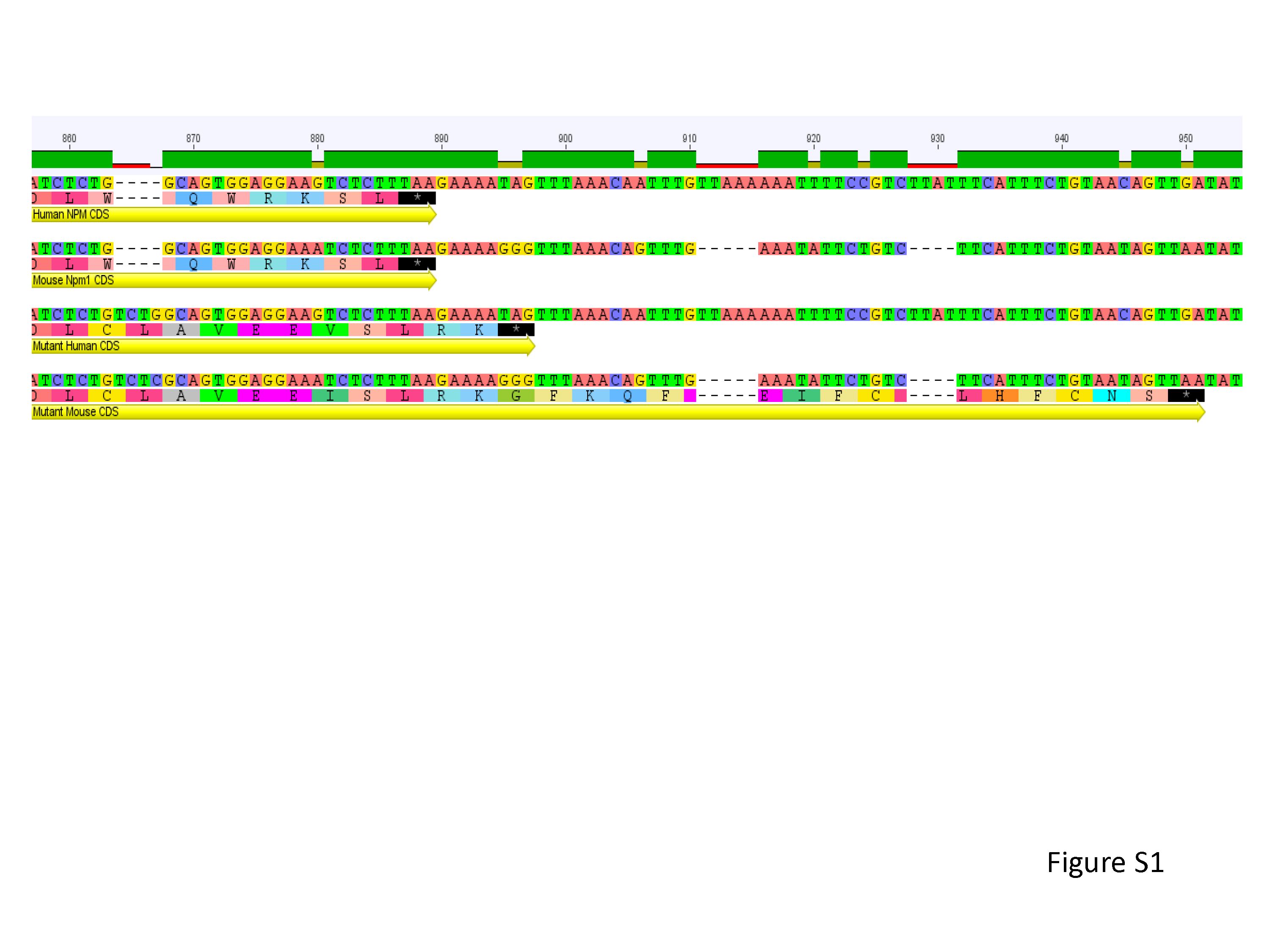

Supplement: Figure S1 — The alignment of nucleotide and protein sequences of wild-type and mutant NPM1 of human and mice. CDS, coding sequence. (TIF) [file pone.0049769.s001.tif]

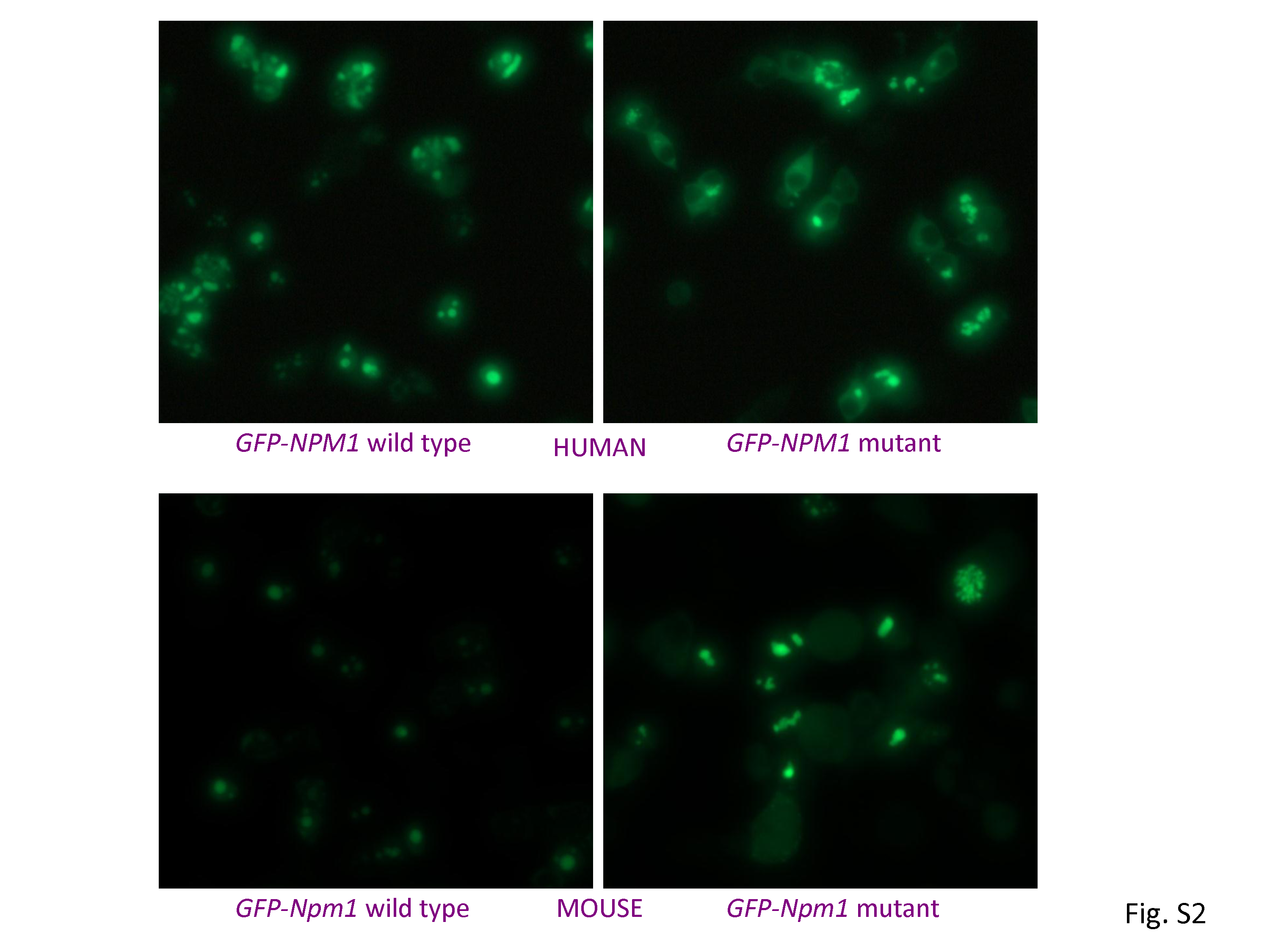

Supplement: Figure S2 — The cytoplasmic localization of mutated nucleophosmin protein from human and mice. Upper panel: GFP fused at the N terminal end of human wild-type NPM1 (left) localizes mainly in the nucleoli as punctate pattern. In contrast, GFP fused with human mutant NPM1 protein mainly resides in cytoplasm, although some appears in the nucleoli. Lower panel: GFP fused at the N terminal end of wild-type mouse Npm1 protein (left) and expected mutant Npm1 protein (right) exhibit similar patterns as the human condition. (TIF) [file pone.0049769.s002.tif]
