# Supplementary material for: A Knock-In Npm1 Mutation in Mice Results in Myeloproliferation and Implies a Perturbation in Hematopoietic Microenvironment
Source: PLoS One. 2012 Nov 30;7(11):e49769. doi: 10.1371/journal.pone.0049769 (PMC3511491; doi:10.1371/journal.pone.0049769)
Supplement: Table S2 — Parameters and settings of GSEA in this Study. (DOCX) [file pone.0049769.s004.docx]

**Supplemental Table S2**

**Table S2. Parameters and Settings of GSEA in this Study****

| Parameter | Setting |
| --- | --- |
| Permutation Type | Gene sets |
| Number of Permutations | 1,000 |
| Enrichment Statistics | Weighted |
| Metric for Ranking Genes | T-test/ Ratio of classes^*^ |
| Collapsing Probes into Genes | Median of probes |
| ^*^ For small sample sizes, e.g. mouse microarray data, ratio of classes is adopted for gene ranking, while for larger ones, t-test is employed to order genes based upon statistical significance.  ^**^ Parameters not listed here are set by default in GSEA software. | |
