# Supplementary material for: A Knock-In Npm1 Mutation in Mice Results in Myeloproliferation and Implies a Perturbation in Hematopoietic Microenvironment
Source: PLoS One. 2012 Nov 30;7(11):e49769. doi: 10.1371/journal.pone.0049769 (PMC3511491; doi:10.1371/journal.pone.0049769)
Supplement: Table S3 — Summary of mouse and human gene expression data in gene set enrichment analysis. (DOCX) [file pone.0049769.s005.docx]

**Supplementary Table 3. Summary of mouse and human gene expression data in gene set enrichment analysis**

| Gene set* |  | Gene set size | |  | NES | |  | P** (FDR*** q) | |
| --- | --- | --- | --- | --- | --- | --- | --- | --- | --- |
|  |  | mouse | human |  | mouse | human |  | mouse | human |
| CXCL12  signature |  | 257 | 308 |  | 1.73 | 1.72 |  | <0.001 (0.064) | <0.001 (0.005) |
| CXCR4  signature |  | 157 | 190 |  | 1.66 | 1.67 |  | <0.001 (0.066) | <0.001 (0.005) |

*Only gene sets with significant enrichment (P<0.01) are listed here. Complete summary of GESA is included in Supplemental Table S4.

^**^ Normalized p-value based on 1,000-time permutation tests on gene sets.

***FDR, false discovery rate

Abbreviations: NES, normalized enrichment scores, denoting the degree of enrichment in gene expression profiles of the samples.
